# Skin Cancer in Residential Care Facilities: A Scoping Review of Risk, Diagnosis, Psychosocial Impact, and Management

**DOI:** 10.7759/cureus.102154

**Published:** 2026-01-23

**Authors:** Emily M Garelick, Morgan Ulczak, Jamie Hedrick, Hannah Welp, Katheryn Bell, Elena Myalo

**Affiliations:** 1 Dermatology and Osteopathic Medicine, Philadelphia College of Osteopathic Medicine, Philadelphia, USA; 2 Dermatology, St. George’s School of Medicine, St. George's, GRD; 3 Dermatology, Oasis Dermatology Group, McAllen, USA; 4 College of Osteopathic Medicine, Lincoln Memorial University DeBusk College of Osteopathic Medicine, Harrogate, USA; 5 Dermatology, Indiana University School of Medicine, Indianapolis, USA; 6 College of Osteopathic Medicine, Lake Erie College of Osteopathic Medicine, Erie, USA

**Keywords:** assisted-living facilities, long-term care facilities, nursing homes, older adults, psychosocial factors, skin cancer, supportive care interventions, systematic barriers

## Abstract

The older adult population is rising, resulting in a larger number of adults residing in nursing homes. Skin cancer is most commonly diagnosed in older adults. Yet, these individuals often face limited access to advanced dermatologic care, in addition to unique risk factors such as inadequate sun protection and reduced mobility. This review aims to map the current evidence on skin cancer risk and diagnosis, as well as prevention strategies among older adults residing in nursing homes. A search was conducted on PubMed using the algorithm: ((nursing home) OR (assisted living) OR (elderly living facility)) AND (skin cancer), where 269 papers were retrieved. Inclusion criteria included full-text primary sources published from 2015 to 2025. Exclusion criteria included duplicates, reviews, animal studies, and studies not about skin cancer in nursing home settings. Title review narrowed the search to 38 papers. Following abstract screening, five final papers were included. A comprehensive review of the included studies demonstrated that there is a high prevalence of skin cancer among nursing home residents. There were a total of 4785 participants among the five papers, and skin cancer prevalence varied from 15 to 40% across studies. Nearly half of the diagnosed cases were basal cell carcinoma, a quarter were squamous cell carcinoma, and the final quarter was unspecified. Factors that contribute to the prevalence of skin cancer include age, immobility, incontinence, and a lack of appropriate sun protection. Additionally, systematic barriers, such as limited access to specialized dermatologic services, the absence of standardized screening practices, and limited staff training, increase the risk of delayed diagnosis and inadequate management of skin cancer in nursing home patients. Skin cancer in older adults, particularly those in long-term care, is also shaped by psychosocial challenges such as personality traits that influence distress, reduced quality of life, and delays in seeking treatment, highlighting the importance of early detection and supportive care interventions. To improve outcomes for this vulnerable population, there is a need to implement routine skin assessments, strengthen staff education on dermatologic care, and increase timely specialist referrals for proper detection and treatment of skin cancer.

## Introduction and background

As the global population continues to rise, a growing number of older adults reside in long-term care facilities. Within the next 15 years, there is a predicted 47% increase in care demand [1]. Within these settings, dermatologic care remains an underrecognized concern, despite its significant impact on residents’ quality of life [2]. The incidence of both melanoma and non-melanoma skin cancers (NMSC), which primarily includes basal cell carcinoma (BCC) and squamous cell carcinoma (SCC), continues to rise among the elderly population [3]. Melanoma arises from acute, intermittent sun exposure leading to malignancy of melanocytes and is very important for early detection to reduce morbidity and mortality [4]. NMSC occurs due to exposure to ultraviolet radiation (UV), which leads to malignant keratinocyte transformation leading to a non-reactive cutaneous inflammatory response [5]. With NMSC, mortality is low although morbidity commonly impacts patients due to involvement of sun-exposed areas of the skin [5]. BCC and SCC commonly occur in areas of sun exposure; additionally, BCC tends to be slow-growing [5]. Metastasis is more rare in BCC, unlike SCC, which has a 1-10% likelihood of invasion [5]. Within the entire global population, an estimated 2-3 million NMSC cases are diagnosed annually [6]. In the United States, it is projected that by age 65, at least half of the population will have experienced at least one incidence of skin cancer ​[7]​. Elderly adults within hospitals, short or long-term care facilities have a cancer prevalence between 0.6 and 13.5% of patients ​[3]. While genetic predisposition plays a role, environmental factors such as chronic ultraviolet (UV) exposure, chemical carcinogens, and tobacco use are strongly linked to increased skin cancer risk ​[3]. Given these trends, there is a clear need to expand access to dermatologic care within residential facilities, yet numerous barriers persist. 

Older adults often face complex health challenges, including multiple comorbidities, cognitive decline, and limited mobility, which reduce engagement in routine care and hinder access to dermatology services. Visiting outpatient clinics may be impractical for many, and in-facility dermatologic consultations remain rare due to time constraints and limited financial incentives for providers [2]. Compounding these challenges is the widespread lack of sun protection in long-term care settings. Cumulative UV exposure is a major risk factor for NMSC [8], and older adults, especially those with sun-sensitive skin, are nearly twice as likely to experience sunburns [9]. While preventative measures like sunscreen, protective clothing, and wide-brimmed hats are available, their use is often inconsistent. Practical issues such as improper sunscreen application, poor coverage, and missed reapplication further reduce their effectiveness [9]. 

Beyond cancer, other dermatologic conditions are prevalent in nursing homes, notably incontinence-associated dermatitis (IAD) [10]. IAD frequently affects residents with limited mobility and results from prolonged exposure to moisture, leading to skin barrier breakdown, inflammation, itching, and pain [10]. Without timely hygiene and skin care, IAD can cause significant discomfort and secondary complications [10]. Residents in long-term care facilities face unique dermatologic challenges posing risks to physical health, but also diminishing their quality of life and psychosocial well-being. 

The burden of dermatologic conditions extends beyond physical health. Visible skin diseases may reduce self-esteem, interfere with social engagement, and diminish quality of life [11]. Residents may experience embarrassment of social withdrawal, while caregivers and peers may unintentionally exhibit bias toward those with visible skin conditions. Personality traits and levels of social support can also influence coping mechanisms [11]. Utilizing validated quality-of-life questionnaires can help healthcare providers better understand the emotional and functional impacts of dermatologic conditions and tailor care accordingly [11]. Physicians should recognize these psychosocial dimensions and engage with patients in a respectful, nonjudgmental manner to foster comfort and dignity.

As the aging population grows, so does the need for high-quality, accessible dermatologic care in long-term care settings. However, reliable data on skin cancer prevalence in this group remains limited. Some studies estimate NMSC rates in nursing home residents as high as 7.2%, with BCC ranging from 3.9% to 14.8% and SCC up to 8% [3,8]. However, these figures are likely underestimates due to underdiagnosis, particularly among residents with cognitive impairment or severe frailty. In many cases, skin lesions go unnoticed until they are advanced, due to provider oversight, institutional constraints, or the residents' inability to report symptoms. This underscores a critical gap in routine assessment protocols and diagnostic follow-up.

Moving forward, improving dermatologic care in nursing homes requires a multifaceted approach. First, integrating regular, structured skin assessments into geriatric evaluations, conducted by trained nursing staff or primary care providers, could greatly increase early detection of suspicious lesions. Second, targeted education for healthcare workers in long-term care should emphasize skin cancer recognition and appropriate referral pathways. Third, preventive strategies such as promoting sun-safe behaviors, ensuring consistent sunscreen application, and optimizing access to shaded outdoor spaces must be prioritized. Finally, addressing the psychosocial burden of skin disease through supportive, patient-centered communication is essential to improving quality of life. As life expectancy increases and more individuals enter long-term care, these strategies will be essential to protecting the dermatologic health and dignity of this vulnerable population.

The purpose of this review is to systematically map and synthesize the current evidence on the prevalence, risk factors, diagnostic pathways, and psychosocial impacts of skin cancer among older adults residing in nursing homes and assisted-living facilities. The primary research question guiding this scoping review follows: What is known from the existing literature about the burden of skin cancer, the barriers to its detection and management, and the psychosocial consequences of the disease in institutionalized older adults? This review aims to characterize the reported prevalence of skin cancer and tumor types in long-term care settings, identify clinical and systemic barriers to timely diagnosis and treatment, and examine how psychosocial factors, such as cognitive impairment, personality traits, and quality of life, influence disease recognition and outcomes. The objective is to identify gaps in the current literature and to inform future clinical practice, research priorities, and policy development related to dermatologic care in nursing homes. This article is a scoping review of primary research studies, designed to provide a broad, structured overview of the available evidence rather than to evaluate intervention efficacy or perform meta-analysis.

This article was previously presented as a meeting abstract at the 2025 DermLink Scholars Inaugural Virtual Research Conference on August 2, 2025.

## Review

Methods

Study Design and Objective

This study was conducted as a scoping review using Preferred Reporting Items for Systematic Reviews and Meta-Analyses for Scoping Review (PRISMA-ScR) guidelines. The objective was to map and summarize the available evidence on skin cancer prevalence, diagnosis, and psychosocial factors among older adults residing in nursing homes and assisted-living facilities. The review was designed to identify knowledge gaps and barriers to dermatologic care in long-term care settings rather than to evaluate treatment efficacy.

Search Strategy

A literature search was conducted in PubMed on May 18, 2025, covering studies published from January 1, 2015, to May 18, 2025. The following search terms were used: ((nursing home) OR (assisted living) OR (elderly living facility)) AND (skin cancer).

Additional terms including “psychology,” “mental health,” and “quality of life” were used to identify contextual psychosocial literature. PubMed was used as the sole database because it provides comprehensive coverage of peer-reviewed dermatology, oncology, and geriatric medicine literature and was sufficient for mapping the available evidence in this focused clinical area.

Inclusion and Exclusion Criteria

Final inclusion criteria were as follows: (i) Peer-reviewed primary research studies (observational studies, cohort studies, cross-sectional studies, case series, or case reports); (ii) Published between January 1, 2015, and May 18, 2025; (iii) Written in English; (iv) Available as free full-text articles; (v) Included human participants; (vi) Conducted in nursing homes, assisted-living facilities, or long-term care institutions; (vii) Reported original data on the diagnosis, prevalence, management, or outcomes of skin cancer.

Exclusion criteria were: (i) Review articles, systematic reviews, meta-analyses, editorials, and commentaries; (ii) Animal or laboratory-based studies; (iii) Studies not conducted in long-term care or institutionalized elderly populations; (iv) Studies not specifically related to skin cancer; (v) Conference abstracts without full-text articles.

Data Extraction

After screening and applying the inclusion criteria to the studies obtained from the relevant databases, researchers organized the information on a data log that included the title, type of review and year, inclusion/exclusion criteria, sample size and age, location, diagnosis, and treatment. The final outcomes were documented on a Google Docs spreadsheet. With the information organized, a thorough discussion of each article was conducted to determine whether it fit the inclusion criteria.

The initial search elicited 269 articles based on the outlined search criteria. After removing 231 which were outside of the included date range and non-free full text articles, an additional 33 were filtered out as they were secondary sources. The final five articles selected involved patients living in nursing homes or assisted living facilities with skin cancer.

Psychosocial studies were reviewed separately for contextual relevance because they did not meet the setting-specific inclusion criteria, but informed interpretation of quality-of-life and distress related to skin cancer. More than one psychosocial study was reviewed to ensure consistency of themes.

Quality and Risk of Bias Assessment

As this study was a scoping review, formal risk of bias assessment was not required and was therefore not performed, consistent with PRISMA-ScR guidelines. The purpose of this review was to describe the extent and nature of the available evidence rather than to grade study quality. However, study design and sample size were documented to allow readers to contextualize the strength of the evidence.

Results

The initial PubMed search identified 269 records. After removal of articles published outside the 2015 to 2025 timeframe and those without free full-text availability, 231 records were excluded. Thirty-eight articles underwent title and abstract screening, and 33 were subsequently excluded because they were reviews, secondary sources, or did not meet the predefined inclusion criteria. Five studies met all eligibility requirements and were included in the final scoping review. These five studies comprised two multicenter observational studies, one retrospective cohort study, one prospective teledermatology study, and one case report, representing a total of 4,785 nursing home and assisted-living residents across Europe and Australia.

**Figure 1:**
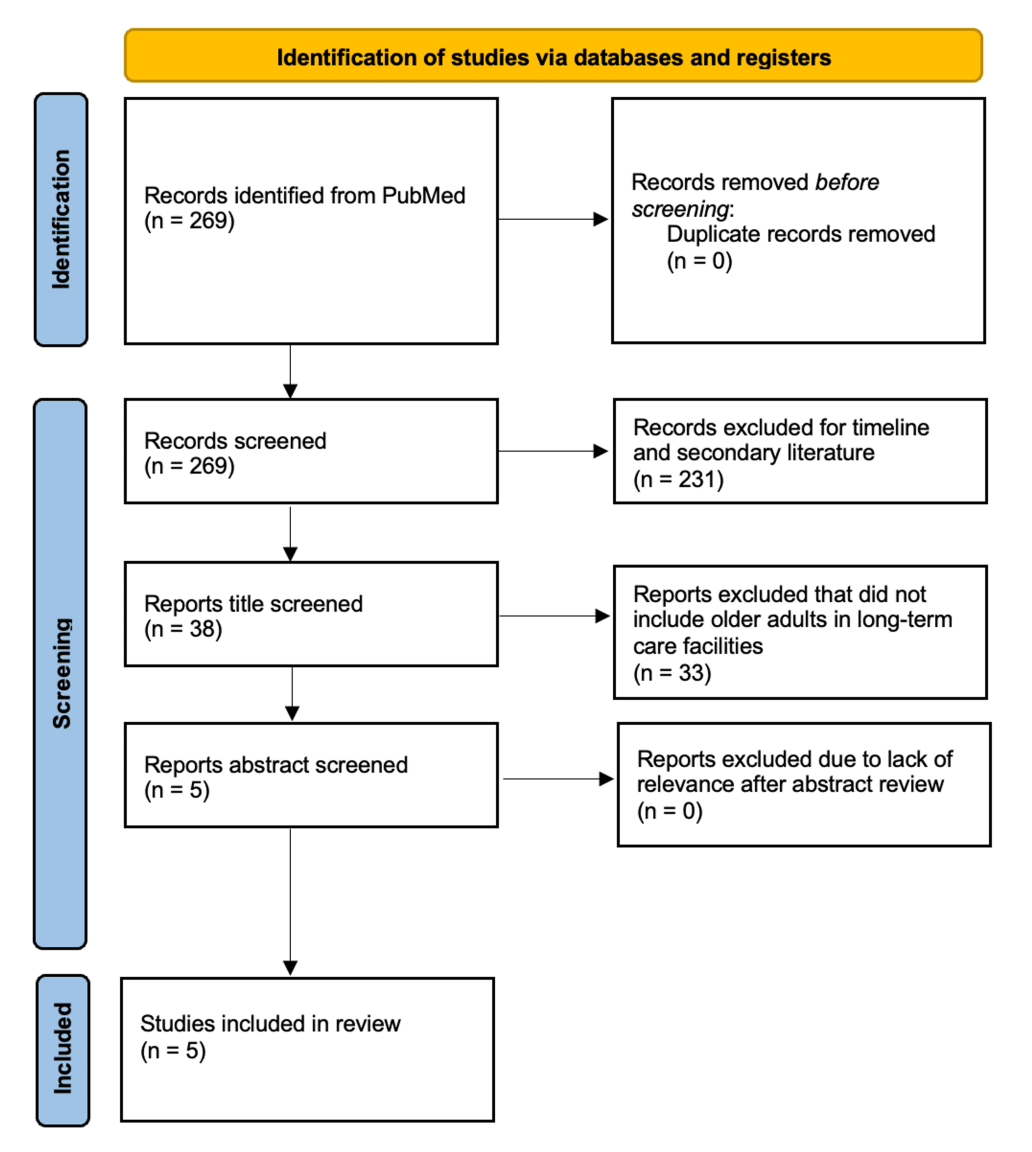
PRISMA 2020 flow diagram for scoping literature review of skin cancer in older adults living in long-term care facilities This figure outlines the study selection process for a scoping literature review of skin cancer in older adults residing in long-term care facilities. The initial PubMed search generated 269 papers, which was narrowed to a final five studies after thorough review. PRISMA: Preferred Reporting Items for Systematic Reviews and Meta-Analyses

Across the final five articles reviewed (Figure 1), the prevalence of skin cancer ranged from 15% to 40%, with BCC comprising approximately 50% of cases and SCC around 25% (Table 1). A multicenter prevalence study by Akdeniz et al. evaluated 223 adults aged 65 and older in 10 institutional long-term care facilities in Berlin, Germany, a population of fair-skinned individuals at elevated risk for both melanoma and NMSC [3]. The study found that nearly one in five nursing home residents had premalignant actinic keratosis lesions, the most commonly observed skin condition in the cohort (21.1%, 95% CI 16.2 to 26.9). NMSC was identified in 16 residents (7.2%, 95% CI 4.5 to 11.3), with one patient diagnosed with lentigo maligna. No cases of malignant melanoma were reported. This strongly suggests a high burden of skin malignancies in this aging, fair-skinned population.

**Table 1:** Included articles following scoping review of skin cancer in older adults living in long-term care facilities.

Study	Sample Size	Participants	Location	Study Concept	Cancer Type	Key Findings
Akdeniz, 2019 [3]	223	Gender: 67.7% F; Mean age: 83.6; Other major characteristics: 36.3% former smokers, 12.7% current smokers	Berlin	Multicenter prevalence study	Non‑melanoma skin cancer (NMSC): 16 residents (~7.2%); Basal cell carcinoma (BCC): 15 residents (6.7%); Cutaneous squamous cell carcinoma (cSCC): 1 resident (0.4%); No malignant melanoma cases detected (0%)	NMSC: 7.2%; Melanoma: 0%; Recommend: regular dermatologic screening in nursing homes & greater involvement of primary care professionals
Liuu, 2019 [12]	214	Gender: 63% F; Mean age: 89.7; Other major characteristics: 42% cognitive impairment	France	Retrospective descriptive study	Skin cancer: 56, but did not specify which type	Skin cancer: 26.2% Older residents: less cancer staging & fewer cancer-specific treatments; Younger residents: more likely to get follow-up care & interventions; recommend: better cancer detection training, improved staging protocols, & tailored treatment depending on age
Wildiers, 2019 [13]	4262	Gender: not reported; Mean age: 87; Other major characteristics: limited	Belgium	Prospective multicenter cohort study	Skin cancer: 2, but did not specify which type	Skin cancer: less frequently than expected compared to the older population. Many suspected cancers were not biopsied or treated (due to frailty). Recommend: earlier retrospective reports & careful assessment of diagnostic and treatment strategies
Klösters, 2022 [14]	85	Gender: 63.3% F; Mean age: 85; Other major characteristics: 89.1% polypharmacy, 66.6% not legally competent to make own medical decisions	Germany	Prospective observational study	Basal cell carcinoma (BCC): 3 patients; squamous cell carcinoma (SCC): 2 patients. Other suspicious skin lesions or precancerous conditions: reported, but exact numbers not specified.	Recommend: Teledermatology as a triage tool (improve access to dermatologic care for nursing home residents & optimize resource use)
Lobo, 2020 [15]	1	Gender: 100% F; Mean age: 80; Other major characteristics: dementia and former smoker	Australia	Case report	SCC	Challenges in diagnosing & managing large, neglected skin cancer in elderly nursing home residents Recommend: improved skin cancer screening, timely intervention, & multidisciplinary care

The study also found a correlation between actinic keratosis and male sex, and a correlation between NMSC and both smoking and female sex [3]. While literature has supported a link between smoking and SCC, the relationship between smoking and BCC remains inconclusive. Nonetheless, the data suggested an overall increase in risk in the development of NMSC in patients who reported a history of smoking [3]. Although certain lifestyle factors may contribute to skin cancer risk, once irreversible factors such as genetic predisposition and cumulative UV exposure have taken effect, early dermatologic intervention is essential. Prompt treatment of premalignant lesions can help prevent progression to invasive cancer, especially in elderly populations who often have limited access to routine dermatologic care compared to younger individuals. In the Netherlands, for instance, only 30% of surveyed dermatologists reported ever seeing patients within a nursing home setting, with 51.4% of those visits involving premalignant lesions [3].

Liuu et al. conducted a retrospective study to analyze the prevalence of skin cancer occurring after age 75 in a cohort of 214 residents across 45 French nursing homes [12]. The median age was 90 years (IQR 87-93; range 76-104), and 63% of patients were female [12]. The prevalence of cancer diagnosed after age 75 was 8.4% ± 1.1. Most residents had a median nursing home stay of two years (IQR 1-4; range 0-25). Notably, 71% of cancer cases were diagnosed within the five years before the study, with the majority occurring after age 85 (53%), and 37% identified following admission to a nursing facility [12]. Skin cancer was the most frequently diagnosed cancer type (26%), followed by cancers of the digestive tract (18%) and breast (18%) [12]. Interestingly, the overall cancer prevalence among nursing home residents was lower than that observed in the general community, raising the question of whether this reflects a true decrease in disease incidence or a potential underdiagnosis in institutionalized settings. Cognitive impairment was the most prevalent comorbidity, affecting 42% of participants, and 44% were rated as highly dependent based on the Autonomie Gérontologique Groupes Iso-Ressources (AGGIR) scale used by the French government [12]. These findings highlight the importance of identifying residents who could benefit from early diagnostic evaluations and timely treatment. Comprehensive geriatric assessments with a multidisciplinary team across nursing home staff may help guide this management, especially in patients with cognitive impairment who may not be able to advocate for themselves or adequately express their concerns.

Wildiers et al. conducted a prospective, multicenter cohort study across 39 Belgian nursing homes, following a total of 4,262 residents over the course of one year to identify new cancer diagnoses [13]. Two of the nine nursing home residents were reported to have skin cancer, though the distinction between melanoma and nonmelanoma was not specified. Overall, five new suspected cancer diagnoses and four instances of cancer progression were reported, corresponding to an annual incidence rate of 123 per 100,000 nursing home residents [13]. It is important to note that cancer events occurred at a much lower frequency than expected from population-based data. For comparison, data from the Netherlands Cancer Registry reported a cancer incidence of 2,566 per 100,000 person-years among individuals aged 75 and older in 2015 [13]. These findings support previous reports that cancer events are rare in frail older adults, and diagnostic or therapeutic interventions in this patient population are lacking [16]. A major challenge in geriatric oncology research is selection bias [16]. Clinical trials often exclude older and frail patients, often due to ethical concerns, logistical limitations, or presumed inability to adhere to treatment protocols [16]. As a result, many suspected cancers in nursing home residents are never biopsied or treated [17]. Decisions to forego diagnostic workups or active management are frequently influenced by patient frailty, family preferences, or institutional policies, further contributing to underdiagnosis and undertreatment in this population [17].

Klösters et al. distinguished their study from prior research by specifically examining the role of telemedicine as a diagnostic tool for dermatologic consultations in nursing home residents [14]. This prospective multicenter observational study evaluated the reasons for dermatology consultations, including factors predicting the added value of live consultations. Malignant skin lesions were found to be the most frequent reason for telemedicine consultations (161/270; 59.6%). Dermatologists determined that in-person consultations provided added value over teledermatology in 67.8% of cases (183/270) [14]. However, it is important to note that telemedicine visits can help prioritize the identification of cases that truly require in-person evaluation [14]. This can facilitate preoperative planning to ensure that in-person visits have maximum efficiency [14]. This added benefit was reported to include consultations for skin tumors, cases involving diagnostic or treatment procedures, and consultations where a secondary diagnosis was made. These findings suggest that teledermatology could reduce the need for live consultations in about one-third of cases [14]. Most nursing home dermatology consultations were found to be for inflammatory or infectious conditions, but skin cancers also represented a significant proportion of consultations. Teledermatology can serve as a triage tool to prioritize referrals, optimize resource use, and improve access to dermatologic care for nursing home residents [18]. This is crucial in this patient population due to the limited availability of dermatologists who provide on-site consultations.

Lobo et al. reported the case of an 80-year-old nursing home resident who initially presented for an influenza vaccination but was found to have a large, fungating lesion on the chest [15]. Biopsy confirmed the lesion to be SCC. While there was no evidence of perineural or lymphovascular invasion, histopathological examination revealed a depth greater than 2 cm, placing the tumor in a high-risk category for metastasis [15]. This case report highlighted that large, neglected skin cancers can be a frequent challenge in elderly nursing home residents in terms of both recognition and management. It is suggested that late diagnosis, which contributes to advanced disease presentation, can be due not only to lack of recognition but also to medical comorbidities, denial, mental illness, and self-neglect. These factors highlight the critical need for the implementation of regular skin exams, including skin cancer screening programs in nursing homes, to identify suspicious lesions in their early phases. By integrating these skin checks as part of a regular routine in nursing homes, referrals for appropriate care can be made more promptly. This approach can help reduce delays in diagnosis and prevent the development of large, high-risk skin cancers in this patient population.

Psychosocial Effect

Older adults, particularly those in long-term living facilities, face unique psychosocial challenges in the context of skin cancer. Many residents are unable to fully protect themselves from sun exposure or monitor for new or changing lesions, and cognitive or physical decline often limits their ability to seek medical attention independently [19]. As a result, psychosocial factors such as dependence on caregivers, comorbid illness, and emotional distress become central to their skin cancer experience. The reviewed studies are outlined in Table 2.

**Table 2:** Psychosocial effects of older adults living in long-term care facilities with skin cancer

Study	Population	Psychosocial factor examined	Main Finding	Implication
Ramirez-de los Santos et al., 2021 [20]	Forty-seven Mexican skin cancer patients with a mean age of 66.5 years	Personality dimensions related to anxiety and depression in skin cancer patients	There is a positive correlation between neuroticism and psychoticism with depressive symptoms	Certain personality dimensions can increase vulnerability to depressive symptoms
Shah et al., 2006 [21]	One hundred patients ≥ 65 years with various skin conditions	Quality of life in relation to type and extent of skin disease	Rashes were associated with a worse quality of life	Quality of life assessments can be a helpful tool in patients with skin disease
Alam et al., 2011 [22]	Nine hundred and eighty-two patients undergoing Mohs surgery for nonmelanoma skin cancer	Factors contributing to delayed treatment	Forms of denial are the most common cause of delayed presentation	Education programs may help reduce denial in patients with nonmelanoma skin cancer

Personality dimensions may influence how patients cope with skin cancer. Ramirez-de los Santos et al. found that higher neuroticism and psychoticism scores were associated with greater depressive symptoms in patients with early-stage skin cancer, while extraversion was linked to lower distress [20]. These findings suggest that certain personality dimensions may increase vulnerability to distress, especially in older adults who already face challenges such as dependency, social isolation, and reduced control over their health [20]. 

Shah et al. examined the impact of skin disease on 100 patients aged 65 and older with various skin conditions [21]. Although patients with rashes, such as eczema, xerosis, and psoriasis, had higher anxiety and depression scores than those with skin lesions, those with malignant or premalignant lesions (76% of patients), including BCC, actinic keratoses, and melanoma, still reported psychosocial impacts. Tools such as the Dermatology Life Quality Index (DLQI) and Hospital Anxiety and Depression Scale (HADS) proved useful in quickly identifying those at risk of reduced quality of life or mood disturbances, making them practical additions to clinical assessments [21].

It is also important to investigate potential reasons for delay in treatment which can lead to an increase in size and risk of metastatic spread. Alam et al. investigated reasons for delayed presentation in 982 patients undergoing Mohs micrographic surgery for nonmelanoma skin cancer [22]. It was found that for increased tumor size, patients were more likely to have waited a longer period of time before being seen. It is important to note that tumor size was patient-reported and may have been subject to recall bias. Out of the patients that waited (n=550), the most common reasons included belief of self-resolution (36%), unknown importance of getting checked (24%), unable to secure an earlier appointment (10%), too busy (5%), and self-treatment (5%) [20]. This indicates that the most common cause of delay was denial of the illness, and to counteract this, education programs that emphasize the benefits of early detection can be useful.

Discussion

Skin cancer is highly prevalent among residents of nursing care facilities and remains the most common malignancy in this population [12]. This review aimed to explore the scope of this prevalence and to examine the factors contributing to underdiagnosis and delayed detection. Only five studies in the PubMed search that were published in the past decade met the inclusion criteria. Notably, 80% of the studies focused on European populations, primarily involving individuals with fair skin tones [3,10,11,14,16]. Furthermore, all included studies either did not distinguish between NMSC and melanoma or focused exclusively on NMSC as the primary cancer type [3,12,13,16,18]. While this review unveiled the limited number of recent studies and the limitations of these studies on skin cancer in older adults in residential facilities, further studies can provide longitudinal data, expand upon the representation of diverse racial and ethnic populations across the globe, and research the prevalence and impact of melanoma in elderly populations in care facilities. This review was limited to only one database and future reviews can expand upon more databases beyond PubMed. 

Multiple barriers hinder timely diagnosis and treatment of skin cancer in nursing home residents. Systemic challenges such as lack of structured screening protocols, transportation difficulties, and limited access to dermatologic care are compounded by patient-specific factors including cognitive impairment, comorbidities, and personal reluctance to seek care [22]. Due to advancing age, comorbidities, and frailty, patients and caregivers may decide against undergoing diagnostic procedures such as skin biopsy or treatment for skin cancer in favor of more palliative measures [2,13]. Caregivers who are not trained to recognize skin cancers may treat them as ulcers with wound care and delay diagnosis and appropriate treatment [18]. Proper and timely recognition of lesions, access to dermatological care, and routine care and prevention are needed to address the number of NMSC in this population.

Formal training on suspicious lesions, changes in clinical practice, implementing more formal policies for dermatological care, and instituting more preventive measures can address the underdiagnosis, delayed detection, and prevalence of NMSC due to the recognized systemic barriers. Training all nursing facility care providers and clinical staff on how to recognize concerning lesions can improve earlier skin cancer detection and improve outcomes by catching lesions earlier on [3]. For example, detecting actinic keratoses through routine follow-ups can help to prevent malignant transformation. Digital in-facility mobile screening via teledermoscopy and teledermatology through self or caregiver-led skin exams may be a way to address diagnosis and initial dermatological consult transportation issues [16,23]. Referrals to dermatology can be streamlined through creating a pathway to navigate all of the systemic constraints when it comes to nursing home residents being able to access specialist care through initial teledermatology visits or through educating clinical providers on dermatological care and when to refer out [2,23,24]. Implementing more preventive care and measures can also help to address this high prevalence of NMSC in the elderly nursing home population. The use of sun-protective clothing, applying sunscreen, providing shade, and UV protection on windows can help to prevent photodamage in this susceptible population [7]. Reinforcing skin protective measures can reduce the risk of further skin damage in this population. 

There is a high prevalence of skin cancer in residents of nursing care facilities and the older adult population, which contributes to psychosocial challenges. Many older patients are unable to fully protect themselves from sun exposure, monitor for new or changing lesions, or independently seek medical attention due to cognitive or physical decline. Consequently, psychosocial factors such as caregiver dependence, comorbid illness, and emotional distress play a central role in their skin cancer experience. Individual personality traits can influence how older adults cope with skin cancer. Higher levels of neuroticism have been associated with increased anxiety and depressive symptoms, while traits such as extraversion may be protective and linked to better psychological adjustment. These personality-driven differences in coping may affect emotional well-being and engagement with treatment in elderly patients with early-stage skin cancer [20]. Skin cancer in older adults is also known to negatively impact quality of life [22]. Delays in diagnosis and treatment, often due to lack of awareness, denial, or misconceptions about the lesion’s severity, can exacerbate these outcomes, leading to larger lesions and more invasive interventions, further increasing psychosocial stress [22]. These findings highlight the importance of early detection, not just for clinical outcomes, but also for maintaining emotional and psychological well-being. Targeted education programs for both residents and caregivers are essential to overcome misconceptions and emphasize the value of timely care.

## Conclusions

Older adults residing in nursing homes face a high, but often overlooked, risk of skin cancer worsened by barriers to prevention, diagnosis, and treatment. Despite rising rates of skin cancer in this population, routine screenings and timely referrals remain rare, leading to delayed care and poorer outcomes. System-wide solutions are urgently needed, such as standardized screening, staff training, improved access to dermatologic expertise, and consistent sun protection. Future research should incorporate more databases, include diverse populations, and assess long-term clinical and psychosocial outcomes. Prioritizing preventative skin cancer in long-term residential settings can reduce disease burden and improve quality of life for older adults.
